# Computational Prediction of the Combined Effect of CRT and LVAD on Cardiac Electromechanical Delay in LBBB and RBBB

**DOI:** 10.1155/2018/4253928

**Published:** 2018-11-14

**Authors:** Aulia K. Heikhmakhtiar, Ki M. Lim

**Affiliations:** Department of IT Convergence Engineering, Kumoh National Institute of Technology, Gumi 39177, Republic of Korea

## Abstract

Two case reports showed that the combination of CRT and LVAD benefits the end-stage heart failure patients with prolonged QRS interval significantly. In one of the reports, the patient had the LVAD removed due to the recovery of the heart function. However, the quantification of the combined devices has yet to be conducted. This study aimed at computationally predicting the effects of CRT-only or combined with LVAD on electromechanical behaviour in the failing ventricle with left bundle branch blocked (LBBB) and right bundle branch blocked (RBBB) conditions. The subjects are normal sinus rhythm, LBBB, RBBB, LBBB with CRT-only, RBBB with CRT-only, LBBB with CRT + LVAD, and RBBB with CRT + LVAD. The results showed that the CRT-only shortened the total electrical activation time (EAT) in the LBBB and RBBB conditions by 20.2% and 17.1%, respectively. The CRT-only reduced the total mechanical activation time (MAT) and electromechanical delay (EMD) of the ventricle under LBBB by 21.3% and 10.1%, respectively. Furthermore, the CRT-only reduced the contractile adenosine triphosphate (ATP) consumption by 5%, increased left ventricular (LV) pressure by 6%, and enhanced cardiac output (CO) by 0.2 L/min under LBBB condition. However, CRT-only barely affects the ventricle under RBBB condition. Under the LBBB condition, CRT + LVAD increased LV pressure and CO by 10.5% and by 0.9 L/min, respectively. CRT + LVAD reduced ATP consumption by 15%, shortened the MAT by 23.4%, and shortened the EMD by 15.2%. In conclusion, we computationally predicted and quantified that the CRT + LVAD implementation is superior to CRT-only implementation particularly in HF with LBBB condition.

## 1. Introduction

Heart failure (HF) plays a major role in the number of death worldwide [1]. Thus, the study of heart diseases including cardiac arrhythmia, which progressively leads to HF condition, is very important. Two types of cardiac therapy devices are commonly used to treat patients with cardiac disease: cardiac resynchronization therapy (CRT) and left ventricular assist device (LVAD). CRT is considered as a valuable treatment for patients with dyssynchrony HF with QRS interval >120 ms and left ventricular ejection fraction (EF) <35% [2, 3]. A number of studies showed significant benefits of using CRT. CRT synchronizes systolic function [4–6], restores heart structure [7, 8], and improves symptoms and the quality of life of the patients identified by the improvement of exercise endurance [3, 8–12]. However, 30% of patients with HF failed to benefit from the CRT [9]. Hu et al. [13] described that three major responses could be obtained from the CRT treatment: resynchronization of the intraventricular contraction, efficient ventricular preloading by a properly timed atrial contraction, and reduction of mitral regurgitation. One of the study findings was that resynchronization of the intraventricular contraction itself did not necessarily lead to stroke work improvement. However, the synchronization of the atrioventricular firing time was essential.

LVAD supports the ventricular pumping via mechanical unloading for weakened heart. LVAD was initially used as a bridge to transplantation for patients with end-stage disease [14, 15]. However, because the availability of heart donors is very limited, LVAD is now used as destination therapy as it lasts for years [16–18]. LVAD reverses the damaged ventricle and recovers myocardial functionalities by repairing left ventricular (LV) mass [19, 20], heart chamber size [20, 21], improves mitral filling [22], and induces cardiomyocyte hypertrophy regression [21, 22]. The use of LVAD also increased the quality of life of the patients [23–25]. Previously, we observed the effect of LVAD on the electromechanical delay (EMD) under mild and severe HF conditions [26]. The results showed that the LVAD not only increased the EF but also shortened the EMD under the mild HF and even better under severe HF conditions. The comparison study of the symptomatic relief of CRT and LVAD by Delgado et al. concluded that the use of both devices could synergistically improve cardiac functions for severe HF treatment [27].

In 2011, a case report article stated that the CRT supported the restoration of end-stage HF in a 15-year-old patient who underwent LVAD implantation [28]. The CRT shortened the septal to posterior wall motion delay from 146 ms to 104 ms, and overall, it backed up the hemodynamic improvements. After such improvement, the patient successfully had the LVAD removed. In another case report article, the CRT and LVAD cooperatively restored the cardiac functions of a 62-year-old patient who had cardiogenic shock and left bundle branch block (LBBB) [29]. The report stated that the combination of CRT and LVAD performed a profound treatment on the weakening heart under LBBB condition. It recovered the heart hypertrophy, and the EF was increased. Based on these reports, we conducted a computational modelling which combined the CRT and LVAD to the failing ventricle with LBBB and RBBB in order to understand the mechanism of the two devices combined to provide such improvement to the heart.

To the best of our knowledge, a computational study that quantifies the effect of combined CRT and LVAD has yet to be conducted. This study aims at predicting, computationally with an electromechanical failing ventricles model, the effects of CRT alone and the combination of CRT and LVAD treatment in patients with LBBB or right bundle branch block (RBBB). We used a well-developed electromechanical ventricular model which had been used to observe different heart conditions, including the prolonged EMD in the dyssynchrony HF condition [30], mechanoelectrical feedback on scroll wave stability [31], and spontaneous arrhythmia in acute regional ischemia [32]. Recently, by using similar electromechanical coupling model, our group revealed the effect of KCNQ1 S140G mutation on arrhythmogenesis and pumping performance [33]. We used computational methods to overcome the measurement limitations and risks of the experimental study. We analyzed seven HF diseases and therapies: (i) normal sinus rhythm, (ii) LBBB, (iii) LBBB with CRT alone (LBBB + CRT), (iv) LBBB with CRT and LVAD combination (LBBB + CRT + LVAD), (v) RBBB, (vi) RBBB with CRT alone (RBBB + CRT), and (vii) RBBB with combined CRT and LVAD (RBBB + CRT + LVAD) conditions.

## 2. Materials and Methods

**Figure 1:**
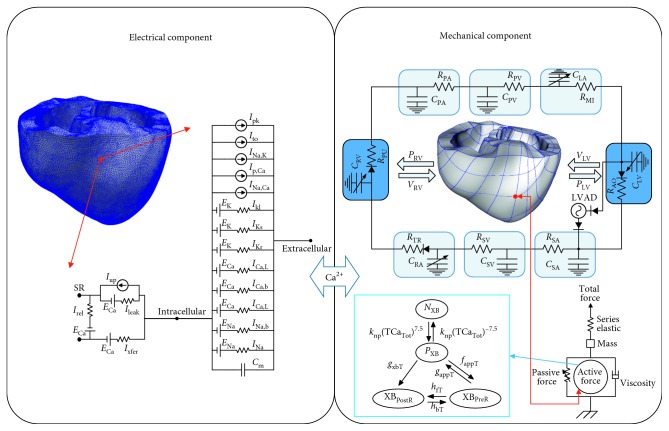
Schematics of the electrical and mechanical elements coupled with transient calcium. Electrical element: it represents the currents, pumps, and exchanger of the ten Tusscher ionic model as explained in equation (1). Mechanical element: a schematic diagram of the finite-element ventricular mechanical model coupled with the circulatory and LVAD models. P_RV_, RV pressure; V_RV_, RV volume; P_LV_, LV pressure; V_LV_, LV volume; R_PA_, pulmonary artery resistance; C_PA_, pulmonary artery compliance; R_PV_, pulmonary vein resistance; C_PV_, pulmonary vein compliance; R_MI_, mitral valve resistance; C_LA_, left atrium compliance; R_AO_, aortic valve resistance; R_SA_, systemic artery resistance; C_SA_, systemic artery compliance; R_SV_, systemic vein resistance; C_SV_, systemic vein compliance; R_TR_ tricuspid valve resistance; C_RA_, right atrium compliance; R_PU_, pulmonary valve resistance.

We followed an existing well-developed electromechanical failing ventricle model with the fibers and laminar sheet structures based on diffusion tensor magnetic resonance imaging [34–37]. In this study, our electromechanical model was coupled with circulatory systems and LVAD models similarly as Lim et al. with additional Purkinje networks compartment to simulate LBBB and RBBB conditions [38]. The electromechanical model consisted of electrophysiological and myofilament dynamics model coupled by Ca^2+^ transient. The LVAD function included the circulatory systems, which connected to the 3D ventricular mechanics.  Figure 1 shows a full schematic of the system we used in this study.

**Figure 2:**
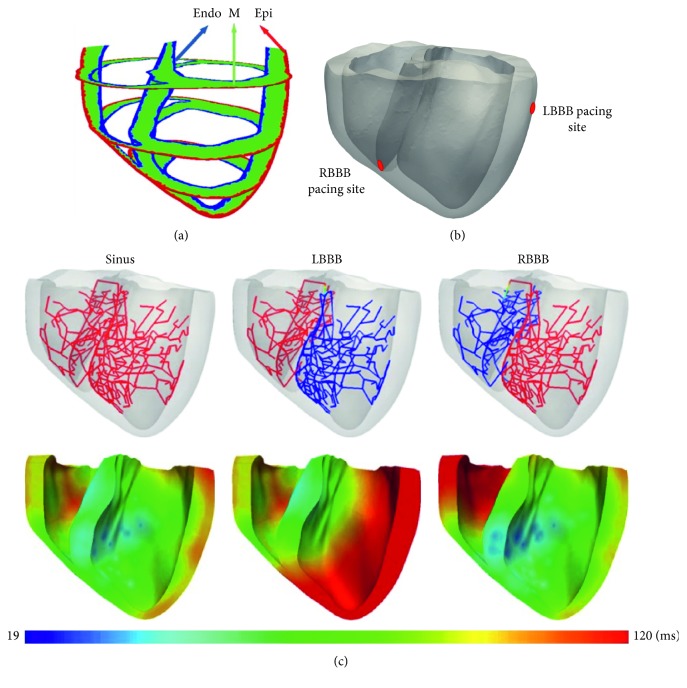
(a) The heterogeneous mesh that has the characteristics of endocardial, midmyocardial, and epicardial cells. (b) The CRT pacing site for the LBBB heart was placed on the LV free-wall, while that for the RBBB heart was placed inside the RV at the bottom of the septum. (c) The electrical propagation by the Purkinje network in the sinus, LBBB, and RBBB conditions. The electrical activation by the Purkinje networks is indicated in red. In sinus pacing, the Purkinje network delivers the electrical signal to its terminals. In the LBBB condition, the Purkinje network delivered the signal only to the right network in the RV area. In the RBBB condition, the Purkinje network delivered the signal only to the left network in the LV area. CRT, cardiac resynchronization therapy; LBBB, left bundle branch block; LV, left ventricular; RBBB, right bundle branch block; RV, right ventricular.

The electrical component was a failing ventricular mesh with 241,725 nodes and 1,298,751 tetrahedron elements. The electrical mesh has the characteristics of realistic heart compartment, which consists of endocardial, midmyocardial, and epicardial cells following the ten Tusscher et al. human ventricular tissue model [39]. The line mesh type representing Purkinje networks was mapped onto a 3D ventricle chamber as well at the endocardial region (Figure 2(c)).

The Purkinje networks induced the electrical activation sequences of sinus rhythm (normal), LBBB, and RBBB (Figure 2(c)). The CRT pacing site of the LBBB was placed at the LV free-wall, while the CRT pacing site of the RBBB was placed at the RV endocardial apex as shown in Figure 2(b). The electrical stimulation was first induced at the root node of Purkinje fiber model, which propagates to the terminals, hence stimulating the ventricular tissue. The electrical propagation in the Purkinje can be described by solving a one-dimensional wave propagation equation and triggered the ventricular activation [40].

The electrical propagation signal represents an ion exchange across the myocyte as described by ten Tusscher et al. [39]. The electrophysiological phenomenon for the single cell can be described as follows:

(1) $$\begin{array}{c} \frac{dV}{dt}=\frac{-I_{\text{ion}}+I_{\text{stim}}}{C_{\text{m}}}, \end{array}$$

where *V* represents the voltage difference of intracellular and extracellular, *t* represents time, *I*_ion_ represents the sum of all transmembrane ionic currents, *I*_stim_ represents the current if an external stimulus is applied, and *C*_m_ represents the cell capacitance per unit of surface area. *I*_ion_ consists of major ionic current as follows:

(2) $$\begin{array}{c} I_{\text{ion}}=I_{\text{Na}}+I_{\text{K}1}+I_{\text{to}}+I_{\text{Kr}}+I_{\text{Ks}}+I_{\text{CaL}}+I_{\text{NaCa}}+I_{\text{NaK}}+I_{\text{pCa}}+I_{\text{pK}}+I_{\text{bCa}}+I_{\text{bNa}}, \end{array}$$

where *I*_Na_ is fast inward Na^+^ current, *I*_K1_ is inward rectifier K1 current, *I*_to_ is transient outward K^+^ current, *I*_Kr_ is rapid delayed rectifier K^+^ current, *I*_Ks_ is slow delayed rectifier K^+^ current, *I*_CaL_ is L-type inward Ca^2+^ current, *I*_NaCa_ is Na^+^/Ca^2+^ exchanger current, *I*_NaK_ is the Na^+^/K^+^ pump current, *I*_pCa_ and *I*_pK_ are sarcoplasmic plateau Ca^2+^ and K^+^ currents, *I*_bCa_ is background Ca^2+^ current, and *I*_Na,b_ is background Na^+^ current.

To represent the electrical propagation through the conduction in 3D spatial, the cardiac tissue in this case could be described by the combination of the Equation (1) with the cellular resistivity (*ρ*) and surface-to-volume ratio (*S*) in *x*, *y*, and *z* directions, respectively. This phenomenon can be described by the following partial differential equation:

(3) $$\begin{array}{c} \frac{dV}{dt}=\frac{-I_{\text{ion}}+I_{\text{stim}}}{C_{\text{m}}}+\frac{1}{\rho_{x}S_{x}C_{\text{m}}}\frac{\partial^{2}V}{\partial x^{2}}+\frac{1}{\rho_{y}S_{y}C_{\text{m}}}\frac{\partial^{2}V}{\partial y^{2}}+\frac{1}{\rho_{z}S_{z}C_{\text{m}}}\frac{\partial^{2}V}{\partial z^{2}}. \end{array}$$

The mathematical equation for calcium dynamics for coupling the electromechanical simulation was also described by ten Tusscher et al. [39]:

(4) $$\begin{array}{c} I_{\text{leak}}=V_{\text{leak}}\left({\text{Ca}}_{\text{sr}}-{\text{Ca}}_{\text{i}}\right),\\ I_{\text{up}}=\frac{V_{\text{maxup}}}{1+K_{\text{up}}^{2}/{\text{Ca}}_{i}^{2}},\\ I_{\text{rel}}=\left(a_{\text{rel}}\frac{{\text{Ca}}_{\text{sr}}^{2}}{b_{rel}^{2}+{\text{Ca}}_{\text{sr}}^{2}}+C_{\text{rel}}\right)\;dg,\\ {\text{Ca}}_{\text{ibufc}}=\frac{{\text{Ca}}_{i}\times {\text{Buf}}_{c}}{{\text{Ca}}_{i}+K_{\text{bufc}}},\\ \frac{d{\text{Ca}}_{\text{itotal}}}{dt}=\frac{-I_{\text{Cal}}+I_{\text{bCa}}+I_{\text{pCa}}-2I_{\text{NaCa}}}{2V_{c}F}+I_{\text{leak}}-I_{\text{up}}+I_{\text{rel}},\\ {\text{Ca}}_{\text{srbufsr}}=\frac{{\text{Ca}}_{\text{sr}}\times {\text{Buf}}_{\text{sr}}}{{\text{Ca}}_{\text{sr}}+K_{\text{bufsr}}},\\ \frac{d{\text{Ca}}_{\text{stotal}}}{dt}=\frac{V_{\text{C}}}{V_{\text{sr}}}\left(-I_{\text{leak}}+I_{\text{up}}-I_{\text{rel}}\right), \end{array}$$

where *I*_leak_ represents the calcium released from the sarcoplasmic reticulum (SR) into the cell. *I*_up_ represents the calcium pumping to restore the calcium again back to the SR. *I*_rel_ represents the calcium-induced calcium release current, *d* is the activation gate of *I*_rel_, which was the same with *I*_CaL_. Ca_itotal_ is the total calcium inside the cell, which consists of Ca_ibufc_, the calcium buffer inside the cell, and Ca_i_, the free calcium inside the cell. Accordingly, the Ca_srtotal_ is the sum of calcium in the SR, which includes Ca_srbufsr_, the calcium buffer, and Ca_sr_, the free calcium inside the SR.

A Ca^2+^ transient serves as an input to the cell myofilament model representing the generation of active tension within each myocyte in which an ODE set and multiple algebraic equations describe Ca^2+^ binding to troponin C, cooperatively between regulatory proteins, and cross-bridge cycling. Ventricular contraction results from the active tension generation represented by the myofilament dynamics model described by Rice et al. [41]. Ventricular deformation is described by the equations of passive cardiac mechanics, with the myocardium being orthotropically hyperelastic, and nearly incompressible material with passive properties defined by an exponential strain-energy function. Simultaneous solutions of the myofilament model equations to those representing passive cardiac mechanics on the finite-element mesh constitute cardiac contraction. Considering the isometric contraction, we assumed that the sarcomere length (SL) is 0 at the initial value, *d*SL*/dt* = 0. To measure the isotonic contraction, the *SL* is solved using the following ordinary differential equation (ODE):

(5) $$\begin{array}{c} \frac{d}{dt}SL=\frac{{\text{Integral}}_{\text{Force}}+\left({\text{SL}}_{0}-\text{SL}\right)\times \text{viscosity}}{\text{mass}}, \end{array}$$

the viscosity and mass are described in Figure 1 at the mechanical compartment, and Integral_Force_ is the sum of the normalized force integrated toward time:

(6) $$\begin{array}{c} {\text{Integral}}_{\text{Force}}=\int_{0}^{t}\left(F_{\text{active}}\left(x\right)+F_{\text{passive}}\left(x\right)-F_{\text{preload}}-F_{\text{afterload}}\left(x\right)\right)\;dt, \end{array}$$

where the *F*_active_(*x*) is the active force and the *F*_passive_(*x*) is the passive force. *F*_preload_ is the constant force at the resting length of the initial sarcomere length, and *F*_afterload_ is the force during the isotonic or isometric contraction. Thus, *F*_afterload_ is expressed as follows:

(7) $$\begin{array}{c} F_{\text{afterload}}\left(x\right)=\text{KSE}\times \left(x-{\text{SL}}_{0}\right). \end{array}$$

We calculated the ATP consumption by integrating the myofilament model in one cycle proposed by Rice et al. from each node [41]. In the myofilament model, the ATP consumption rate (*E*) is the outcome of cross-bridge detachment rate (*g*_*xbT*_) and the single overlap fraction of thick filaments (SOVF_Thick_):

(8) $$\begin{array}{c} E=g_{xbT}\times {\text{SOVF}}_{\text{Thick}}. \end{array}$$

To construct an integrated model of an LVAD-implanted cardiovascular system, we added a compartment of LVAD function between LV and systemic arteries in the circulatory system based on Kerckhoffs et al. [42] as described previously by Lim et al. [38]. Briefly, the LVAD component was modelled as a flow generator with a specific mean flow rate of 3 L/min. Constant-flow conditions were used to simulate the continuous LVAD with the inlet at the LV apex and the outlet at the ascending aorta.

For the simulation protocol, first we simulated the electrical model with the Purkinje delivering the signal representing sinus rhythm, LBBB, or RBBB until the steady state was achieved. We set the conduction velocity by 60 cm/s and the basic cycle length by 600 ms. Here, we incorporated the dyssynchrony HF conditions (LBBB and RBBB) using CRT-alone or combined CRT and LVAD. We then used Gaussian point for the interpolation of transient Ca^2+^ from the electrical simulation as the input to the mechanical simulation. To model the HF condition, we multiplied the constant of passive scaling in the strain-energy function by 5 times to stiffen the myocardium. The mechanical model was simulated for 20 seconds to reach the steady state. We compared the ATP consumption rates and tension activation during end-systolic volume (ESV) and the strain during end-diastolic volume (EDV) by integrating the information of them from each node. EMD was defined as the time interval between mechanical activation time (MAT) and electrical activation time (EAT). MAT was identified when the local strain was shortened to 10% before its maximum, while EAT was identified when the myocyte started to depolarize, which in our case was −50 mV [30].

## 3. Results and Discussion

### 3.1. Electrophysiological Simulation Results

**Figure 3:**
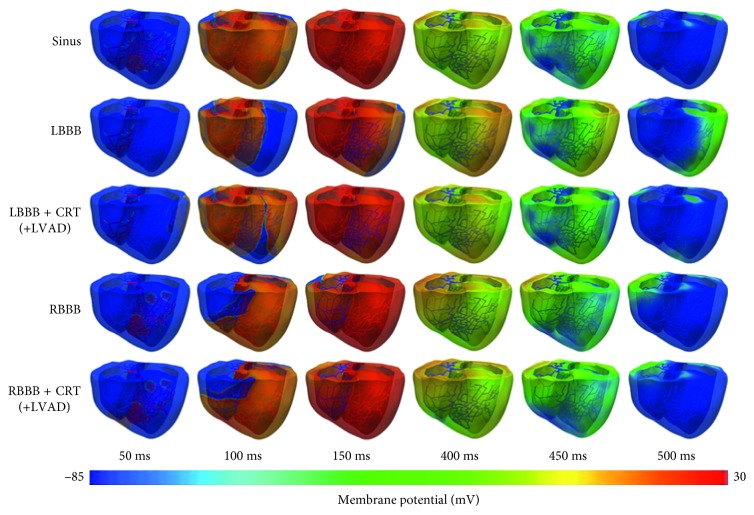
The membrane potential propagation of the sinus pacing, LBBB, LBBB + CRT, RBBB, and RBBB + CRT models. For the LBBB + CRT + LVAD and RBBB + CRT + LVAD models, we used the same electrical activation sequence as the LBBB + CRT and RBBB + CRT models, respectively, because the LVAD was incorporated into the mechanical computation. LBBB, left bundle branch block; CRT, cardiac resynchronization therapy; RBBB, right bundle branch block; LVAD, left ventricular assist device.

Figure 3 shows the membrane potential propagation in the normal sinus rhythm (sinus), LBBB, LBBB + CRT, LBBB + CRT + LVAD, RBBB, RBBB + CRT, and RBBB + CRT + LVAD conditions. The electrical wavelength almost covered the whole ventricle tissue at 100 ms in the sinus condition, and the EAT in the sinus condition was 120 ms (Table 1). The EAT in the LBBB condition was 173 ms, which was the longest among other cases. CRT shortened the EAT in the LBBB condition to 138 ms, which was close to that in the sinus condition. In the RBBB heart, the EAT was 164 ms. CRT shortened the EAT in the RBBB heart to 136 ms, which was close to the sinus condition as well.

**Figure 4:**
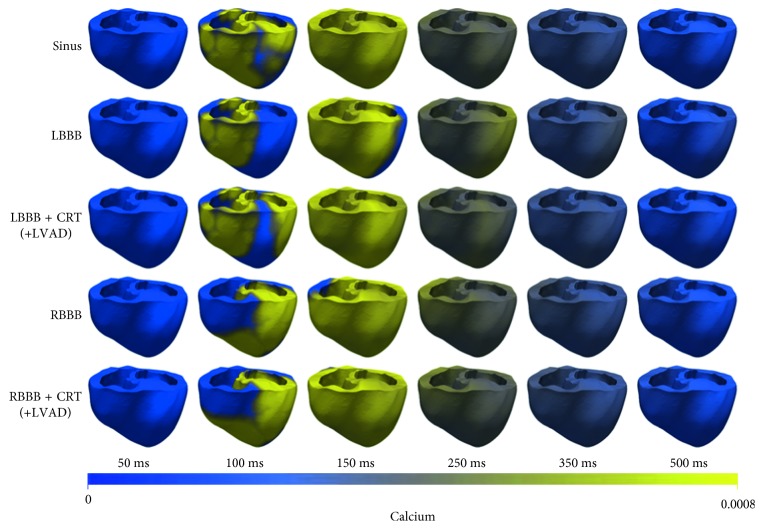
The Ca^2+^ propagation sequence following the membrane potential activation sequence in all cases. LBBB, left bundle branch block; CRT, cardiac resynchronization therapy; LVAD, left ventricular assist device; RBBB, right bundle branch block.

Figure 4 shows the calcium activation sequences. The calcium activation sequence followed the electrical activation sequence for each case. Compared to the membrane potential, which deactivated after 450 ms, the Ca^2+^ deactivation occurred at 250 ms. CRT fastened the Ca^2+^ activation throughout the ventricle in the LBBB condition but insignificantly in the RBBB condition due to the CRT pacing site.

### 3.2. Cardiac Mechanics Simulation Results

**Figure 5:**
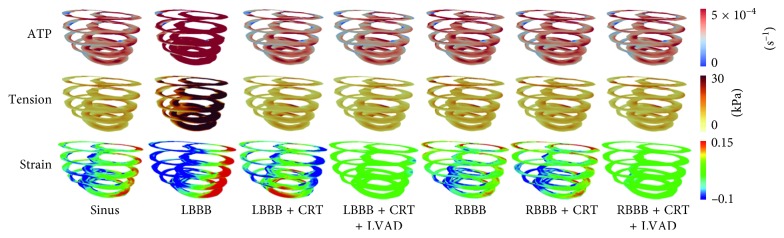
The 3D transmural distribution of ATP, tension, and strain in all cases. The snapshot for the ATP and tension was taken at the end-systolic volume, while the strain snapshot was taken at the end-diastolic volume time. ATP, adenosine triphosphate; LBBB, left bundle branch block; CRT, cardiac resynchronization therapy; LVAD, left ventricular assist device; RBBB, right bundle branch block.

Figure 5 shows the 3D ATP consumption rate, tension, and strain transmural distribution in the sinus, LBBB, LBBB + CRT, LBBB + CRT + LVAD, RBBB, RBBB + CRT, and RBBB + CRT + LVAD conditions. We pick one node at the LV free-wall as representative to compare the values of them. Overall, the ATP consumption rate and tension in the LBBB condition had the highest values, 3.3 and 3.5 times larger than those in the normal sinus condition, respectively. However, the CRT decreased the ATP consumption rate and tension in the LBBB condition to be 6% and 7% lower than those in the normal sinus condition. Furthermore, CRT and LVAD reduced the ATP consumption rate and tension in the LBBB condition to 10% and 12% lower than those in the normal sinus condition, respectively. The ATP consumption rate and tension in the RBBB condition were 8% and 9% higher than those in the control condition. The CRT reduced the ATP consumption rate and tension by 4% and 5%, respectively. CRT and LVAD reduced the ATP consumption rate and tension by 9% and 11% in the RBBB condition, respectively. The strain under the LBBB condition was 80 times larger than that in the normal condition (notified by major red region in the LV free-wall). The CRT reduced the strain in the LBBB 56 times lower than that in the normal sinus condition. CRT significantly restored the total strain under the LBBB condition with the pacing site at the LV free-wall. However, under LBBB + CRT + LVAD, the total strain was only eight times lower than that in the sinus condition. The continuous LVAD altered the overall strain in the ventricles. The total strain in the RBBB condition was 84 times larger than that in the normal condition. In the RBBB + CRT model, the strain activation was 122 times larger. In the RBBB + CRT + LVAD model, the strain activation was 23 times lower than that in the normal condition.

**Figure 6:**
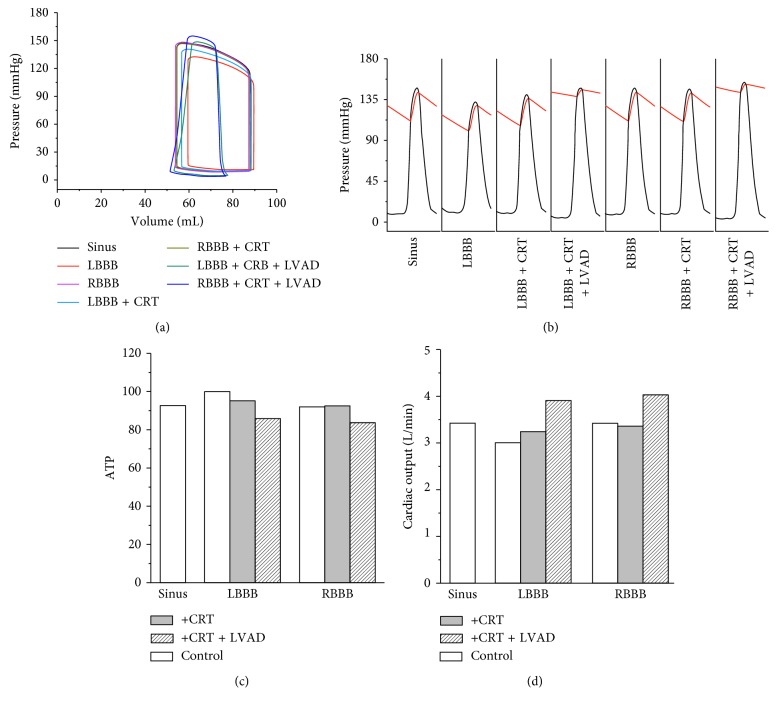
(a) LV pressure-volume loop, (b) LV pressure and aortic pressure, (c) ATP consumption rate, and (d) cardiac output of all cases. LV, left ventricular; LBBB, left bundle branch block; CRT, cardiac resynchronization therapy; LVAD, left ventricular assist device; RBBB, right bundle branch block; ATP, adenosine triphosphate.

Figure 6(a) shows the LV pressure-volume (PV) loop diagram in the sinus, LBBB, LBBB + CRT, LBBB + CRT + LVAD, RBBB, RBBB + CRT, and RBBB + CRT + LVAD conditions. The LV PV-loop in the normal sinus rhythm condition was the same as those in the RBBB and RBBB + CRT conditions. The EDV in the three conditions was 88 mL, ESV was 54.5 mL, and EF was 38%. The LBBB condition had the highest EDV and ESV of 90 mL and 60 mL, respectively. The stroke volume (SV) and EF were 30 mL and 33.4%, respectively. CRT increased the EF up to 36% in the LBBB condition. The EDV, ESV, and SV in the LBBB + CRT condition were 89 mL, 57 mL, and 32 mL, respectively. We did not quantify the EF in models in which LVAD model was incorporated because the PV-loop of the LV was altered due to the LVAD implementation. However, the CO was presented to compare the effects of CRT-alone and combined CRT and LVAD. The complete EDV, ESV, CO, EF, MAT, and EMD data in all conditions are provided in Table 1.

Figure 6(b) shows the LV and systemic artery pressures coloured in black and red lines, respectively. In the normal sinus condition, the peak LV pressure was 148 mmHg. The LV pressure peaked at 358 ms, and the aortic valve was opened for 96 ms. In the LBBB condition, the LV peak pressure was 132.5 mmHg. The LV peak pressure time was prolonged to 407 ms, and the aortic valve was opened for 100 ms. The LV peak pressure in the LBBB + CRT condition was increased to 141 mmHg. The LV peak pressure time was shortened to 364 ms, and the aortic valve was opened for 96 ms. In the LBBB + CRT + LVAD condition, the peak LV pressure was 148 mmHg, the same as that in the normal condition, the LV peak pressure time was 363 ms, and the aortic valve was opened for 62 ms. The combination of CRT and LVAD restored the LV peak pressure and LV peak pressure time in the LBBB condition better than CRT alone. In the RBBB and RBBB + CRT conditions, the LV peak pressures and LV peak pressure time were the same at 148 mmHg and 353 ms, respectively, and the aortic valve was opened for 96 ms, the same as that in the normal condition. The reason was that the electrical activation of the LV was not altered in the RBBB condition. In the RBBB + CRT + LVAD condition, the peak LV pressure and LV peak pressure time were increased to 155 mmHg and 350 ms, respectively, and the aortic valve was opened for 63 ms. This finding shows that the combined CRT and LVAD increased the LV pressure and systemic artery more than the CRT-only implementation.

Figure 6(c) shows the overall ATP consumption rate from one cycle of steady state. In the normal sinus condition, the ATP consumption rate was 93 s^−1^. The ATP consumption rate in LBBB condition was the highest at 100 s^−1^. CRT reduced the ATP consumption rate in the LBBB condition by 5%, while CRT and LVAD reduced the ATP consumption rate by 15%. In the RBBB and RBBB + CRT conditions, the ATP consumption rates were the same as that in the normal condition, 93 s^−1^. However, with CRT and LVAD support, the ATP consumption rate in the RBBB condition was decreased by 16% to 84 s^−1^.

Figure 6(d) shows the CO of the seven subjects. CRT increased the CO slightly in the LBBB condition but not in the RBBB condition. With the combination of CRT and LVAD, the CO of the LBBB and RBBB conditions was significantly increased (Table 1).

**Figure 7:**
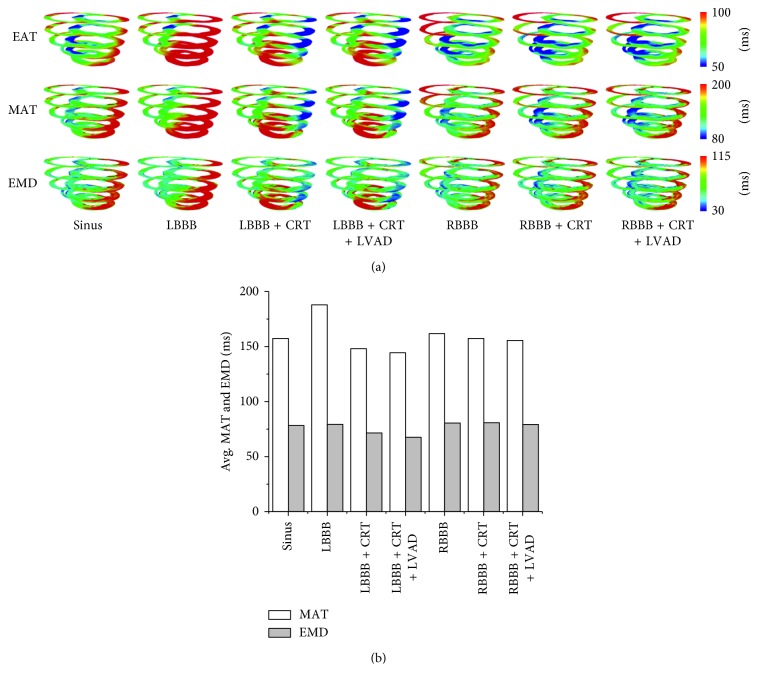
(a) Three-dimensional contour distribution of the EAT, MAT, and EMD and (b) the mean MAT and EMD values in all cases. EAT, electrical activation time; MAT, mechanical activation time; EMD, electromechanical delay; LBBB, left bundle branch block; CRT, cardiac resynchronization therapy; LVAD, left ventricular assist device; RBBB, right bundle branch block.

Figure 7(a) shows the 3D contour of EAT, MAT, and EMD, while Figure 7(b) shows the MAT and EMD of all cases. As shown in Figure 7(a), the activation sequence of MAT and EMD was identical to the EAT. The MAT and EMD values in the normal condition were 157 and 78 ms, respectively. In the LBBB condition, the MAT and EMD values were the greatest at 188 ms and 79 ms, respectively. CRT shortened the MAT and EMD to 148 ms and 71 ms in the LBBB condition. Furthermore, the MAT and EMD in the LBBB condition were further shortened with the combined CRT and LVAD to 144 and 67 ms, respectively. This finding showed that combination of CRT and LVAD performed better than CRT alone despite having the same EAT. In the RBBB condition, the MAT and EMD values were 162 and 80 ms, respectively. In the RBBB + CRT condition, the mean MAT and EMD values were reduced slightly to 157 and 81 ms, respectively. In the RBBB + CRT + LVAD condition, the mean MAT and EMD values were reduced to 155 and 79 ms (close to the control condition), respectively. In the RBBB condition, CRT alone only slightly affected the electrical and mechanical responses. However, CRT + LVAD increased CO in the RBBB condition significantly as expected.

In general, we performed a simulation and analyzed seven cardiac diseases and therapy conditions by using an electromechanical ventricular model incorporated with a circulatory systems, CRT, and LVAD models. The models including normal sinus rhythm, LBBB, LBBB + CRT, LBBB + CRT + LVAD, RBBB, RBBB + CRT, and RBBB + CRT + LVAD. The major findings of this study are as follows:

1. CRT shortened the longest EAT by 20.2% in the LBBB condition and 17.1% in the RBBB condition. CRT shortened EMD by 10.1% in the LBBB condition but did not shorten EMD in the RBBB condition.
2. Combination of CRT and LVAD treatment shortened EMD more than CRT alone in the LBBB condition (15.2%) and shortened EMD in the RBBB condition (1.3%).
3. CRT reduced the ATP consumption by 5% as well as the tension and strain in the LBBB condition. CRT also slightly increased the LV peak pressure by 6% and increased the CO by 0.2 L/min in the LBBB condition. However, CRT-alone did not affect these mechanical responses in the RBBB condition.
4. Combination of CRT and LVAD reduced the ATP consumption by 15% in the LBBB condition and by 16% in the RBBB condition. It also increased the LV pressure by 10.5% in the LBBB condition and 5.7% in the RBBB condition as well as the CO up to 4 L/min for both conditions, a degree greater than that in the control condition.

The pacing site at the tissue greatly affected synchronization. Placement at the LV free-wall showed faster activation throughout the chamber since the signal propagated evenly from the midway between the base and apex throughout the LV tissue. However, the pacing site at the RV endocardial apex showed a longer activation time for the electrical signal to propagate to the base.

In the RBBB condition, the electrical activation of the LV chamber was the same as normal sinus despite the electrical activation alteration in the RV. This is the major factor why the mechanical responses of the LV (LV PV-loop, LV pressure, and CO) were also the same as that in normal sinus rhythm. Even the CRT implementation in the RBBB condition did not affect the mechanical responses of the LV chamber. However, we observed significant improvement in CO in the RBBB + CRT + LVAD condition. CRT and LVAD also reduced the ATP consumption and tension and increased the LV and aortic pressure in the RBBB condition.

CRT-alone did not fully restore the mechanical responses under the LBBB condition to normal despite the resynchronization of the EAT, which shortened the MAT and EMD to back normal. On the contrary, the use of combined CRT and LVAD reduced the energy consumption (indicated by the ATP consumption rate), tension, increased the LV pressure, and produced CO by 18% more than that in the HF with sinus rhythm condition. In addition, the LVAD did not fully assist the blood distribution in the LBBB or RBBB condition.

The computational method allowed us to predict the electromechanical phenomenon in the dyssynchrony heart which underwent CRT and LVAD treatment. It is hardly possible to observe some parameters including ATP consumption, tension, MAT, and EMD from the patient's heart of that condition in experimental procedure. This study quantified the electrical activation and hemodynamic responses in several dyssynchrony HF combined with CRT-only, and CRT and LVAD, which has never been conducted previously. As for the clinical impact, the results of this study can be used as reference to generally predict the effect of the combination of CRT and LVAD devices to the HF patients with LBBB. Though, there are some parameters need to be consider deeply by the cardiologist expert.

This computational study has several limitations that need to be addressed. The study did not follow the standard biventricular pacing method [43]. Instead, we used only one CRT pacing site: the RV endocardial apex (for the RBBB condition) or the LV lateral wall (for the LBBB condition). The LV pacing site was not placed at the optimal position as described before [44]. We did not observe or describe the RV mechanical responses in the RBBB condition; instead, we showed the LV responses only. We used LVAD instead of a right ventricular assist device to support the RBBB condition. Because of our limitations, we did not validate the results of our simulation with experimental data. In addition, we did not describe long-term effects such as recovery of cardiac functions by the combined CRT and LVAD, as previously described [28, 29].

## 4. Conclusions

In conclusion, although CRT-alone shortened the MAT and EMD to more than normal in the LBBB condition, the mechanical responses in the LBBB condition were not restored to normal. The combined CRT and LVAD shortened the MAT and EMD more than CRT-alone, restored the hemodynamic, and produced a greater CO than normal in the LBBB and RBBB conditions. Using the combined system, LVAD contributed to the MAT reduction by mechanical unloading, shortened the EMD, reduced ATP consumption, and reduced tension, which contributes to the recovery of the heart shape and function. In short, we computationally predicted and quantified that the CRT + LVAD implementation is superior to CRT-only implementation particularly in HF with the LBBB condition.

## Figures and Tables

**Table 1:** Hemodynamic responses under normal, LBBB, LBBB + CRT, LBBB + CRT + LVAD, RBBB, RBBB + CRT, and RBBB + CRT + LVAD models.

Condition	EDV (mL)	ESV (mL)	CO (L/min)	EF (%)	Longest EAT (ms)	Average MAT (ms)	Average EMD (ms)
Normal sinus rhythm	88	55	3.4	38	120	157	78
LBBB	90	60	3	33.4	173	188	79
LBBB + CRT	89	57	3.2	36	138	148	71
LBBB + CRT + LVAD	—	—	3.9	—	138	144	67
RBBB	87	54	3.4	38	164	162	80
RBBB + CRT	88	55	3.4	38	136	157	81
RBBB + CRT + LVAD	—	—	4	—	136	155	79

## Data Availability

The methods and results data used to support the findings of this study are included within the article.
